# Using early health economic modeling to inform medical innovation development: a soft robotic sock in poststroke patients in Singapore

**DOI:** 10.1017/S026646232200335X

**Published:** 2023-01-11

**Authors:** Yi Wang, Fan-Zhe Low, Yin-Yi Low, Hwa-Sen Lai, Jeong-Hoon Lim, Chen-Hua Yeow, Yot Teerawattananon

**Affiliations:** 1 Saw Swee Hock School of Public Health, National University of Singapore, Singapore, Singapore; 2 Department of Biomedical Engineering, National University of Singapore, Singapore, Singapore; 3 Department of Medicine, Yong Loo Lin School of Medicine, National University of Singapore, Singapore, Singapore; 4 Singapore Institute for Neurotechnology and Advanced Robotics Center, National University of Singapore, Singapore, Singapore; 5 Health Intervention and Technology Assessment Program, Ministry of Public Health, Nonthaburi, Thailand

**Keywords:** early health technology assessment, medical innovation, target product profiles, real-world application, stroke

## Abstract

**Objectives:**

Based on a real-world collaboration with innovators in applying early health economic modeling, we aimed to offer practical steps that health technology assessment (HTA) researchers and innovators can follow and promote the usage of early HTA among research and development (R&D) communities.

**Methods:**

The HTA researcher was approached by the innovator to carry out an early HTA ahead of the first clinical trial of the technology, a soft robotic sock for poststroke patients. Early health economic modeling was selected to understand the potential value of the technology and to help uncover the information gap. Threshold analysis was used to identify the target product profiles. Value-of-information analysis was conducted to understand the uncertainties and the need for further research.

**Results:**

Based on the expected price and clinical effectiveness by the innovator, the new technology was found to be cost-saving compared to the current practice. Risk reduction in deep vein thrombosis and ankle contracture, the incidence rate of ankle contracture, the compliance rate of the new technology, and utility scores were found to have high impacts on the value-for-money of the new technology. The value of information was low if the new technology can achieve the expected clinical effectiveness. A list of parameters was recommended for data collection in the impending clinical trial.

**Conclusions:**

This work, based on a real-world collaboration, has illustrated that early health economic modeling can inform medical innovation development. We provided practical steps in order to achieve more efficient R&D investment in medical innovation moving forward.

## Introduction

Medical innovation is critical for addressing unmet public health needs and often the answer in times of global health crises, like the cases of diagnostic and therapeutic technologies as well as vaccines for COVID-19 (1;2). Worldwide, billions of dollars are invested in medical product development annually (3). However, it is unclear whether all of these medical innovations can deliver the expected outcomes and bring additional health benefits to the society (4;5). Proper guidance is required to maximize the chances of successful health technology development and to enhance the efficiency of the research and development (R&D) system.

Early health technology assessment (early HTA) has gained increasing attention from policymakers and researchers given its potential for guiding the medical innovation development. It embraces all methods used to inform industry and other stakeholders about the potential value of new medical products in development, including methods to quantify and manage uncertainty (6). Various conceptual frameworks have been developed to apply early HTA (7–9). Technical methods have also been proposed to deal with the challenges of conducting studies at the early innovation stages, such as lack of information and high uncertainties (10–13). The appropriate methods to use depend on the stage of the medical innovation and the purpose of the study.

Nevertheless, early HTA is not widely applied among R&D communities. There are several challenges for early HTA including a disconnection between R&D communities and HTA communities; a lack of awareness of early HTA and its benefits among R&D communities; no standard methodological approach and process guidelines for early HTA; and a lack of examples of successful collaborations between HTA researchers and innovators in applying early HTA (6;14).

Many existing hypothetical early-HTA studies without the involvement of innovators show the theoretical steps that innovators and researchers can follow (15–18); however, certain steps may not be feasible in reality given the considerable challenges faced by and concerns of innovators. Early HTA should be integrated into the innovation process which is frequently driven by the urgency of innovators to get the technology into the market, sometimes in a secretive manner (19). Challenges in integrating early HTA into real-world innovation process are, therefore, less well-studied (20). Real-world collaboration between innovators and HTA researchers is important to identify the challenges and bridge the gap.

Several real-world early-HTA studies along the innovation process have been published, but looked at different innovation stages and settings from this present study (21;22). In our study, the innovator was at the stage with a product prototype before conducting its first clinical trial. We used early health economic modeling to address the request by the innovator. While early health economic modeling has been recommended for more than 20 years (23;24), the details of the early health economic modeling are less frequently published and remain an under-researched area (25).

At no time in history has the world ever been in such demand for a health technology to be successfully developed, efficiently delivered and largely impactful than in the current global health pandemic of COVID-19. In this study, we illustrate a real-world collaboration between HTA researchers and innovators in applying early HTA along the innovation process using early health economic modeling. The aim of the early health economic modeling is to help the innovator understand the potential value of the technology and uncover the data and information gap. The objectives of this study are (i) to describe the steps taken during the entire collaboration; (ii) to present the methodological approaches and analyses; and (iii) to conclude the lessons learnt from the collaboration. We aim to use this real-world collaboration as a concrete example to demonstrate practical steps that HTA researchers and innovators can follow when carrying out similar activities.

## Background: medical issues and the technology

Patients with stroke who are unable to walk are at risk of developing deep vein thrombosis (DVT), especially during the period of inpatient stay (26;27). Subsequently, DVT can cause a fatal condition – pulmonary embolism (PE). Intermittent pneumatic compression (IPC) is an inflatable device that can improve venous circulation in the limbs of patients. Evidence of IPC’s effectiveness in reducing DVT is mixed in the literature (27–31). Spasticity is another common stroke complication, which could lead to joint contracture and functional impairment (32;33). Continuous passive motion (CPM), which repeatedly moves a joint through a predefined range, has been used to release joint contractures. In hospital wards, either bedside passive stretching can be applied to the patients regularly or splints can be prescribed to keep the joint stretched to maintain ankle joint mobility. The limitations of the contracture management in the hospital wards are that the passive stretching can be very labor-intensive to apply sufficiently. The effect will be limited by improper donning and further induced discomfort to the patients.

**Figure 1.**
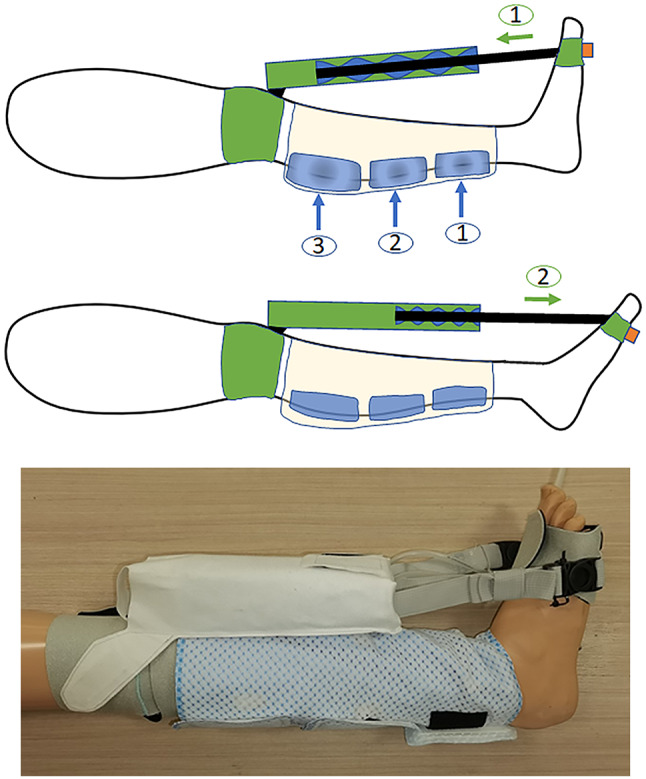
Schematic on the actuation of VACOM system. Sequential compression of the calf with IPC from distal to lateral pockets (labeled blue circles 1 to 3) simultaneously with dorsiflexion of the ankle for the affected limb (labeled green circle 1). On the affected leg, both the IPC and soft actuators deflate simultaneously. The usage of the system requires the clinical users to don the soft robotic actuation device on the immobilized leg affected by stroke and two sets of IPC devices on each separate calf. The actuation mechanism using an electronic pneumatic control box can be separated into two phases. In the first phase, the IPC and soft robotic actuation device on the immobilized leg will simultaneously inflate to compress the calf and dorsiflex the leg for ankle mobilization. In the second phase, the IPC on the mobile leg will be actuated to compress the calf sequentially and promote blood flow. During each phase, while components of the VACOM system are actuated, components on the other limb will be deflated simultaneously to form a complete inflation–deflation cycle. IPC, intermittent pneumatic compression; VACOM, Venous Assistance and Contracture Management.

The medical problem that the clinical team faced for poststroke treatment was that there was not a single device that could simultaneously exercise the immobilized foot to prevent DVT and ankle joint contracture, where prolonged immobilization was a direct cause of both complications. Therefore, the innovator proposed a system named the Venous Assistance and Contracture Management (VACOM), as shown in Figure 1. The system incorporates soft fabric and pneumatic actuators to assist in ankle mobilization, and also conventional IPC device for improving venous circulation in the limbs of patients (34;35).

The benefits of the VACOM device over current standard practice are that first patients can maximize their rehabilitation and recovery poststroke by having a device that provides ankle mobilization while patients are resting in bed. Second, the device reduces the need for use of conventional bulky devices such as the CPM and manpower requirements.

## Methods

### Ethical Approval

This study considered a hypothetical patient group. No human or animal subjects were involved in the study. Hence, ethical approval is not required.

### Study Procedure

The innovator, together with a clinical team directly involved in the development process, approached the HTA researcher aiming to conduct an early HTA on their product prototype before the first clinical trial on the human subjects. Overall key study procedures were summarized in Table 1.

**Table 1.** Summary of the key study procedure

Steps	Timeline	Objectives/Actions	Outcomes/Decisions/Additional comments
First consultation		Understood the stage of the innovation and innovator’s innovation plan for the next steps	The innovator was with a product prototype before conducting its first clinical trial The innovator aimed to begin the first clinical trial in about 3 months The innovator wanted to use the results from this study to inform the design of their coming clinical trials, as well as to aid their future discussions with relevant stakeholders after the clinical trials
		Understood the technology and the relevant background information	Refer to the Background
		Understood the specific requests of the innovator	Refer to the Methods
		Understood the expected timeline of the early HTA work	Complete the early HTA work in 3 months
		Decided the appropriate methods for the early HTA work	Early health economic modeling
		Narrowed down the population, intervention, comparator, outcomes, perspective, and time horizon	Refer to the Methods
		An initial discussion about the details on early health economic models, and the final deliverables	Refer to the Introduction
Signing the contract	Week 0	Signed the contract and started the work	
Between first consultation and second consultation	Week 1– week 7	The innovator shared additional information of the technology and preliminary plan of the coming clinical trial	Refer to the Background
		The researcher conducted the following: Reviewed literature and government reports Built the initial model Collected parameter values	Refer to the Methods
Second consultation	Week 8	Consulted the clinical team and the innovator on the model and model assumptions to finalize the model	Refer to the Methods
		Consulted the clinical team and the innovator on the validity of the information and parameter values collected, and required input on missing parameter values	Refer to Table S1 in the Supplementary Appendix
		Consulted the innovator the scenarios to explore and the analysis to conduct	Refer to the Methods
Between second and third consultation	Week 9–week 13	The researcher conducted the first-round analysis with the following key analyses: cost-utility analysis, both deterministic and probabilistic analyses, to understand the value of the technology; deterministic sensitivity analysis to understand the factors that having high impact on the technology; thresholds analysis to identify the target product profile; value-of-information analysis to understand the need of further research	Refer to the Methods and the Results
Third consultation	Week 14	Presented the results to the innovator and the clinical team	The innovator revised their expectation on the technology The innovator required additional analysis The innovator and clinical team enquired the key messages on the coming clinical trials
After the third consultation		The researcher conducted additional analyses to address the innovator’s comments	Two key groups of the analyses were about price premium and compliance rate
Final deliverables		A final presentation to summarize the key messages and how to use the results from the study A final report	A confidential presentation A confidential report

### Early HTA: Early Health Economic Modeling

The objectives of the innovator were to: (i) estimate the cost-effectiveness of VACOM in its early development stage; (ii) identify the relative importance of key characteristics of VACOM contributing to its value for money; and (iii) understand the data that needs to be collected in the clinical trial and possible future studies. Early health economic modeling was selected to address the needs of the innovator with an emphasize on exploring target product profiles (TPP) and value-of-information (VOI) analysis.

### Study Setup

The population for the study was adult patients with acute ischemic stroke (IS) or hemorrhagic stroke (HA) regardless of lesion size. The patients must develop lower limb weakness measured by Manual Muscle Testing (MMT) ≤ 2 and Modified Ashworth Scale (MAS) ≤ 1+. Patients in the intervention group were assumed to receive VACOM during the inpatient period. Patients in the control group were assumed to receive IPC plus manual ankle movement. The outcome variables included cost, quality-adjusted life years (QALYs), incremental cost-effectiveness ratio (ICER), and net monetary benefit (NMB). A Healthcare system perspective was employed. The time horizon was 1 year.

### Model and Assumptions

**Figure 2.**
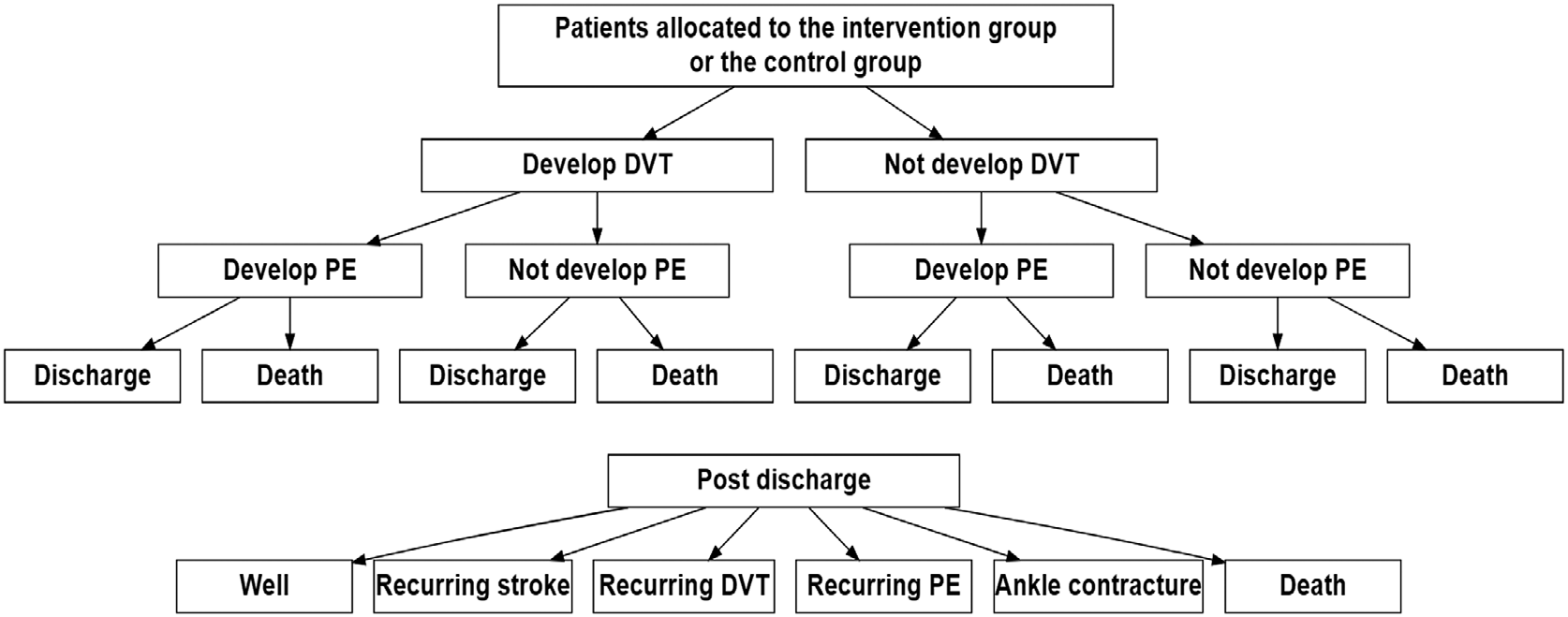
Decision tree. DVT, deep vein thrombosis; PE, pulmonary embolism.

A decision tree model considering the major adverse events was used for the analysis, as illustrated in Figure 2. The timeline was categorized into two periods: inpatient stay and postdischarge. The inpatient stay was assumed to be for 1 month (36). The postdischarge period was from month 2 to month 12. During the inpatient stay, patients may develop DVT and/or PE. For patients who die during the inpatient stay, we assumed they die on the 15th day. For the discharged patients, we considered recurring stroke, recurring DVT, recurring PE, ankle contracture and death. All these adverse events were assumed to happen at the start of the 7th month. More detailed assumptions for the model are presented in the Supplementary Appendix.

### Parameter Values

The clinical effectiveness and cost of VACOM were proposed by the innovator based on their expectation and the results from the previous generation product (35). Epidemiology parameters including incidence rate of DVT, PE, and ankle contracture, mortality rate, and recurrent rate of stroke and complications were derived from the existing literature and government reports (27;37–41). Direct medical cost for stroke patients was collected by the clinical team and derived from the literature, with local studies being prioritized if available (27;42). All the costs were converted into 2019 Singapore dollars (S$). Utility parameters were derived from the literature (43–46). A multiplication formula, for example, 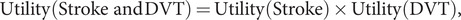 was used to adjust for comorbidities for QALY calculation (47;48). Detailed information on all the parameters can be found in Table S1 of the Supplementary Appendix.

### Analysis

IS patients and HA patients were analyzed separately due to differences in their baseline mortality rates (38). Two scenarios were considered for the analysis; (i) an optimistic scenario where the expected intervention efficacy, that is, the relative risk of ankle contracture for VACOM compared with IPC plus manual ankle movement, was 0.3; (ii) a conservative scenario where the relative risk of ankle contracture for VACOM compared with IPC plus manual ankle movement was 0.9.

#### Cost-Utility Analysis

A cost-utility analysis was conducted to evaluate the cost-effectiveness of VACOM compared to IPC plus manual ankle movement. A ceiling threshold of S$75,000 was considered for the Singapore setting (49).

Probabilistic sensitivity analysis (PSA) was conducted with the results from the analysis presented in the cost-effectiveness acceptability curve (CEAC). One-way deterministic sensitivity analysis (DSA) was conducted with the results presented using tornado plots. NMB was examined to understand the impact of each individual parameter.

#### Threshold Analysis

Threshold analysis was conducted to examine the TPP and maximum additional price that the innovator can charge for VACOM while still being eligible for reimbursement. A few key parameters were selected for this analysis.

#### VOI Analysis

VOI analysis was conducted to understand the value of doing additional research and to help prioritize the parameters to be collected in future research (50;51). First, the expected value of perfect information (EVPI) was calculated using different cost-effectiveness thresholds to determine the opportunity cost of making the wrong decision in adopting VACOM. Second, the expected value of partial perfect information (EVPPI) was calculated to estimate the value in reducing uncertainties for each individual parameter.

## Results

Table 1 summarizes the key collaboration procedures between the innovator and HTA researchers, together with the outcomes and key decisions for each step. Early health economic modeling was identified as the method to address innovator’s concerns during the first consultation. Iterative analysis was conducted which allows the innovator to update their belief and expectation about the technology.

### Value for Money of VACOM

The innovator targets to price VACOM at S$150 extra per patient compared to IPC plus manual ankle movement. We refer this extra price comparing VACOM with IPC plus manual ankle movement as price premium hereafter. VACOM is cost-saving under both the optimistic scenario and the conservative scenario, as shown in Table S2 of the Supplementary Appendix. Figure S1 in the Supplementary Appendix shows the relationship between price premium and ICER, demonstrating the maximum price premium given different ceiling thresholds. Figure S2 in the Supplementary Appendix shows the CEAC.

This price premium of S$150 was revised from the original price premium of S$300 initially set by the innovators. This is because, based on the results from the original analysis using the conservative scenario, the probability of VACOM being cost-effective at the original price premium is only 40–50 percent while this probability is above 90 percent at the revised price premium.

### Relative Importance of Key Parameters

**Figure 3.**
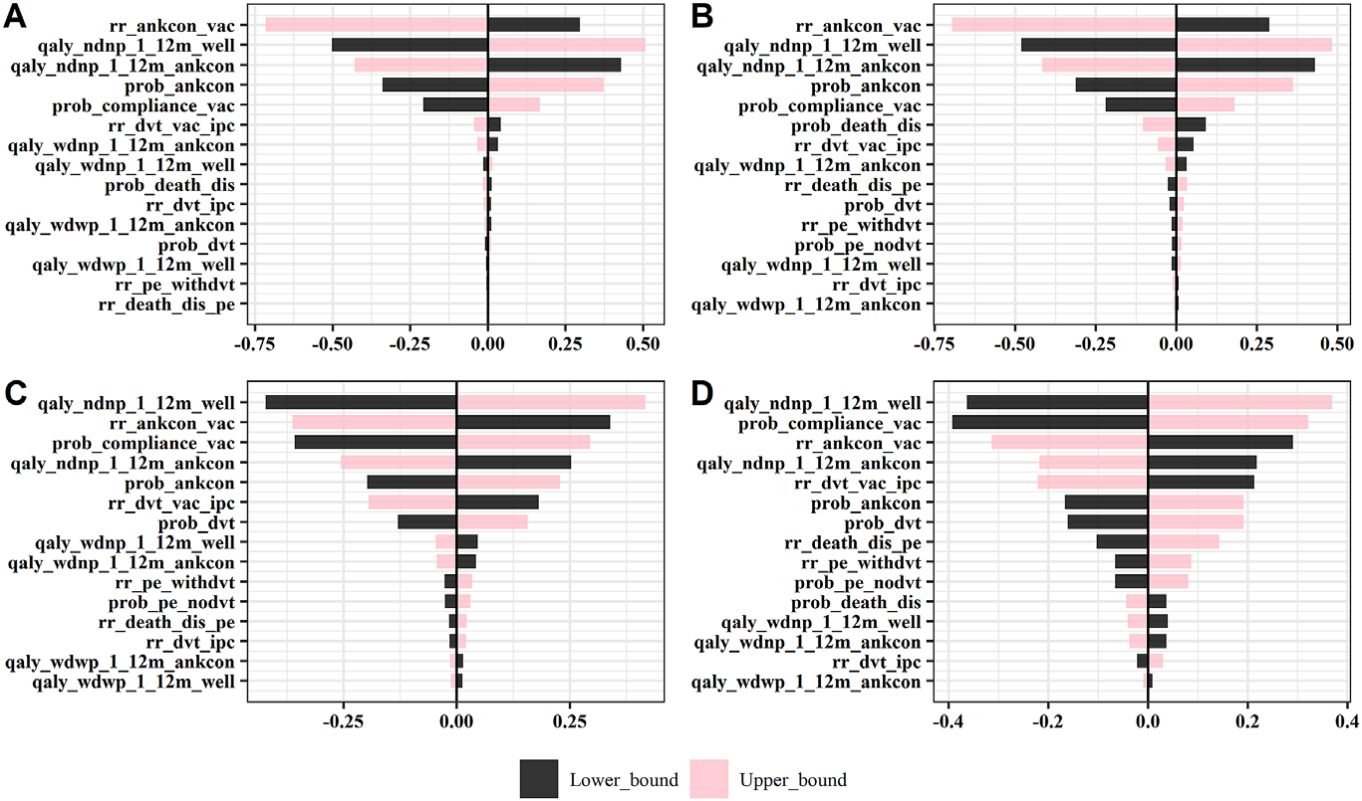
One-way deterministic sensitivity analysis. (A) Ischemic stroke patients under the optimistic scenario; (B) Haemorrhagic stroke patients under the optimistic scenario; (C) Ischemic stroke patients under the conservative scenario; (D) Haemorrhagic stroke patients under the conservative scenario. Readers can refer to Table S1 in the Supplementary Appendix for the full details of each parameter. Explanations for selected top parameters were provided here. rr_ankcon_vac: RR of ankle contracture – VACOM versus IPC + manual ankle movement. qaly_ndnp_1_12m_well: QALY: without DVT without PE during inpatient period, no complication after discharge. qaly_ndnp_1_12m_ankcon: QALY: without DVT without PE during inpatient period, with ankle contracture after discharge. prob_ankcon: Incidence of ankle contracture. prob_compliance_vac: Compliance rate of VACOM. rr_dvt_vac_ipc: RR of DVT: VACOM versus IPC. DVT, deep vein thrombosis; IPC, intermittent pneumatic compression; PE, pulmonary embolism; QALY, quality-adjusted life year; RR: relative risk; VACOM, Venous Assistance and Contracture Management.

**Figure 4.**
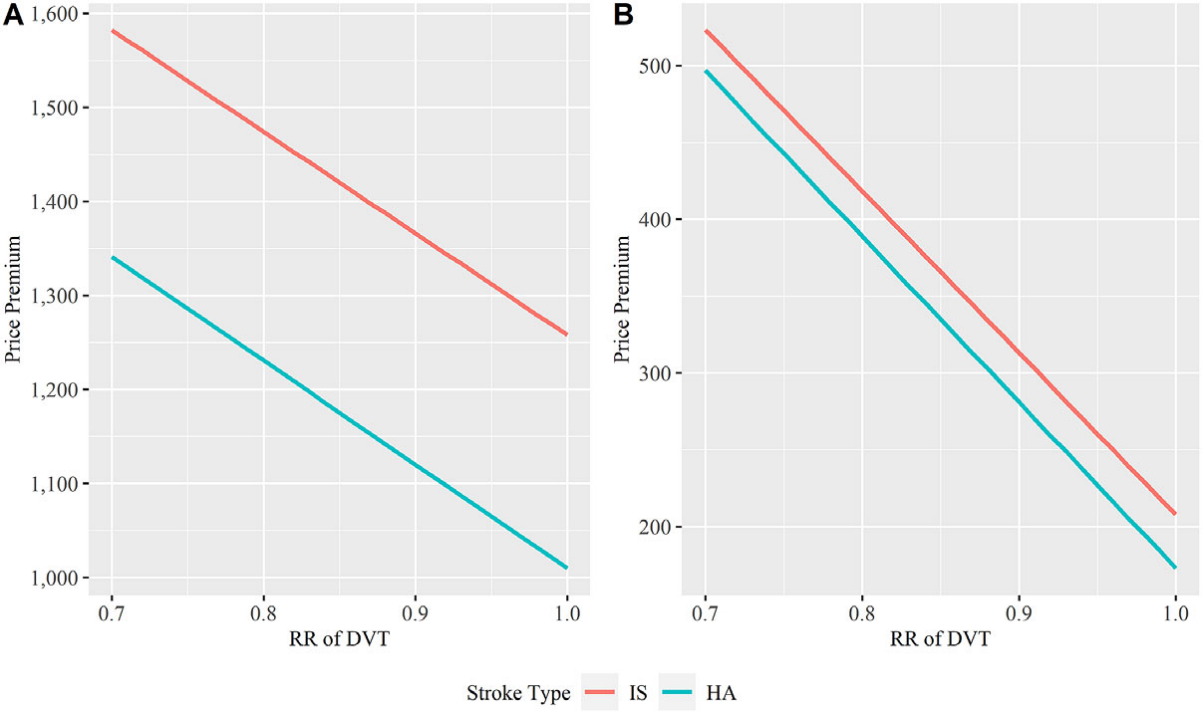
Threshold analysis: Price premium versus RR of DVT. (A) Optimistic scenario; (B) conservative scenario. Price premium is in Singapore dollar. DVT, deep vein thrombosis; HA, hemorrhagic stroke; IS, ischemic stroke; RR, relative risk.

Figure 3 shows the tornado plots from one-way DSA using NMB. The X-axis shows the percentage change in NMB by varying one parameter at a time. The top 15 parameters were shown and ranked in the plot. The innovator can consider these parameters to improve VACOM, identify target patients and appropriate clinical settings. Threshold analysis was utilized for these parameters to understand the maximum price premium by varying the value of these parameters. Figure 4 shows the relationship between the relative risk (RR) of DVT and the price premium that can be charged by the innovator. With a lower RR, which means the VACOM is more effective in preventing DVT, a higher price premium can be charged. Additional results from threshold analysis are presented in Figures S3–S5 in the Supplementary Appendix. The results can be used to understand TPP of VACOM.

### Data and Information Gap

A list of parameters, presented in Table S3 of the Supplementary Appendix was recommended to the innovator to consider in the coming clinical trial and future studies. The parameters were the top parameters from DSA and VOI analysis.

Given the current target price premium, EVPIs were low for VACOM under both optimistic scenario and conservative scenarios, as shown in Figure S6 of the Supplementary Appendix. The overall benefit of additional research is low. More specifically, if the innovator can justify the parameter values used in the analysis in the coming clinical trial, for example, the expected clinical efficacy of VACOM, additional study may not be required. Further, considering the price premium of S$300, EVPI is low under the optimistic scenario compared to the conservative scenario, which suggests a potential trade-off between investing money in improving VACOM and investing money in future research to minimize parameter uncertainty. To help the innovator understand how to prioritize parameters if uncertainties are high, we examined the EVPPI under the scenarios with high EVPI. Price premium of S$1,000 under the optimistic scenario and S$300 under the conservative scenario were considered. The results are presented in Figure S7 of the Supplementary Appendix. The expected cost of a clinical trial, aiming for 100 participants and lasting for 2 years, is around S$1.44 million. Further discussion can be found in the Supplementary Appendix on comparing the cost and benefit of conducting additional research.

## Discussion

Early HTA can inform decisions on medical technology development, design of TPP, allocation of investment in R&D, uncertainty management and future research decisions. However, early HTA is not widely applied among R&D communities due to various barriers. Our work contributes to the literature by showing the experience of a real-world collaboration between HTA researchers and innovators while the technology is in the middle of the development process. A summary of the practical steps is presented in Table 1, in which other HTA researchers and innovators can follow. However, readers should be mindful that this work focused on early health economic modeling to inform medical innovation development based on economic values. HTA is a multidisciplinary process considering social, ethical, legal and organizational dimensions as well. These dimensions are also important to consider in developing and adopting the new technology.

The literature on the theoretical framework of early HTA and methodological development is abundant. While there are established guidelines and checklist for conducting traditional HTA (52), to the best of our knowledge, no such guideline exists for early HTA and early health economic modeling. Innovators are among the target audience of early HTA, which differs from traditional HTA for informing coverage decisions that targets national and hospital decision makers. However, there is little work in the early HTA literature on understanding the needs and concerns of innovators. Without a grasp on innovators’ preferences, the research framework developed by the HTA researchers may not be accepted by innovators. Real-world collaborations between HTA researchers and innovators are important in gauging the needs of innovators as well as to understand how to adapt methods in early HTA setting to avoid undue delay in R&D process while still maintain the quality of the work. These experiences can be harnessed to develop methodological and process guidelines for early HTA, which will be helpful in advancing early HTA collaborations between innovators and HTA researchers.

Early HTA includes many methods for different purposes depending on the innovation stage. While it is ideal to conduct early HTA from the conceptualization stage and throughout the product development process, this requires a strong commitment from innovators and understanding from HTA researchers who are usually conducting assessments on market-approved products for coverage decisions. Practical constraints in conducting technology assessment at the development phases, such as innovation timeline, unavailability of input data and confidential information disclosure, should have special attention in early HTA. In our study, the innovator had a prototype product available; and wanted to understand the quantitative values of their product, cost drivers, profitability and TPP in order to get guidance on their coming clinical trials. They also wanted to prepare materials to facilitate discussion with stakeholders and policymakers on the potential of the innovation. Early health economic modeling is appropriate to help the innovator better understand the innovation’s clinical pathway, service cost drivers and TPP. The model can be iteratively updated using new information from the clinical trials and the comments from relevant stakeholders during future stakeholder consultations. Further, additional analyses on social, ethical and institutional implications can be conducted and combined with the current study.

Different from traditional health economic evaluation, where the baseline scenarios such as patient and product profiles are usually fixed, the baseline scenarios for early health economic modeling are dynamic. HTA researchers need to perform several rounds of analyses and deliberate the intermediate results with innovators. The intermediate results can help innovators form more realistic expectations of the technology and perhaps revisit their plan about technology applications. In this study, the innovator revised the expected price premium in the middle of the study and requested the HTA research team to update the analysis. HTA researchers may also consider multiple baseline scenarios; however, the amount of analysis could be overwhelming without limiting the number of the baseline scenarios. Too many baseline scenarios may also mask the key messages and make the results difficult to interpret by innovators. The design and selection of scenarios should center around the key issues, such as clinical efficacy/effectiveness and price of a new technology as compared to its comparator(s) (13).

Parameters that have a high impact on the value for money of VACOM were suggested to the innovator based on the results from DSA and VOI analysis. In practice, expected value of sample information (EVSI) and expected net benefit of sampling (ENBS) can be used to optimize the clinical trial design such as optimizing the sample size (51). However, EVSI and ENBS analyses were not conducted in this work for the following reasons. First, one of the major aims of the coming clinical trial was to demonstrate the clinical efficacy of the innovation. Innovators wanted to understand the other useful information that can also be collected along the first clinical trial to aid subsequent decision making. Hence, statistical power should be the main consideration for determining sample size at this stage. EVPPI is enough to demonstrate the parameters having high values. Second, calculating EVSI and ENBS require information about data generation process and entire distribution of the parameters. At the stage of the analysis, there was not enough information and many assumptions were required. The results from the EVSI and ENBS will be less accurate. Hence, results from EVSI and ENBS analysis could be more useful after the parameter values being updated from the first clinical trial. More weight should be given to the results from VOI when designing subsequent clinical trials. The HTA researchers should always bear in mind the stage of the innovation and produce the most valuable information to aid decision making.

Threshold analysis was conducted considering the selected top parameters from DSA and examining the relationship between these parameters and the price premium. Similar analysis could be carried out on other parameters pairs. The innovator can use these results to consider whether further R&D can be justified in improving VACOM. Different from the academia where researchers often use ICER, the innovator found NMB concept easier to understand which can be linked to price and profit directly. As such, we presented the results using NMB where possible. Implicitly, this requires an explicit cost-effectiveness threshold.

The results derived are relevant to Singapore and need adaptation in order to extend to other countries. Not only do we used and prioritized information from Singapore in the analysis, but we also used cost-effectiveness threshold relevant for Singapore when calculating NMB. Furthermore, clinical care pathway could also be different in other countries, which could require a different model structure.

Although price or the profit made by the manufacturer from VACOM will be one of the key factors for the innovator when making VACOM available on the market, the HTA researcher recommended the innovator to focus on optimizing the profile of VACOM and identify the right clinical setting and patient group for VACOM. Price itself does not reflect the value of VACOM. The true value of VACOM to the society is in the health benefit that it can bring to the patients and the reduction in medical costs. The information gained from this early HTA exercise can be used for discussion with the national regulatory agency in order to get their agreement on the proposed clinical end point(s) used in the future clinical studies.

One issue that emerged during the work relates to the definition of compliance. The compliance rate was found to have a high impact on the cost-effectiveness of VACOM. While the HTA researcher can simplify the concept and model compliance as binary at a patient level, the clinical definition of compliance is unclear. Hence, the researcher recommended the innovator to incorporate a monitoring system in VACOM. First, given that the meaning of “compliance” is unclear in the clinical setting, the monitoring system can help the innovator understand the minimum required duration and frequency of wear in order to achieve the desired clinical effect. Second, it can help the innovator better estimate the compliance rate. Third, it can help the innovator identify patient group with low compliance rate.

This study has several limitations. First, the model and parameter values used in the study were based on the existing literature, government reports, and the inputs from the clinical team and the innovator. Local electronic medical records could have been used to get more accurate parameter values. Still, HTA researchers need to be mindful of and work within tight timelines. The ethical approval for accessing data may take a few months. Given the medical innovation industry being highly competitive, HTA researchers need to consider how to complete the work in a timely manner to fit innovators’ overall schedule. However, this does not mean the quality of the work was compromised. Researchers need to weigh up these practical challenges when identifying the best and most suitable data, information source, and methods for the study.

Second, the model used is not a comprehensive model capturing the entire caring pathways. For example, other DVT prevention strategies, such as antiplatelet therapy could be used in practice. It is unclear how these other strategies may interact with VACOM. A 5-year time horizon was proposed at the beginning to capture long-term impact. A 1-year time horizon was used due to the following reasons: (i) high-quality long-term data were lacking; (ii) the innovator considered 1-year time horizon more helpful as the planned follow-up period for the coming clinical trial is 6 months; (iii) results from a 1-year model likely bring conservative estimates of the cost-effectiveness of VACOM. Having a model that is acceptable by the decision makers and HTA agencies is important. The clinical team’s involvement in this study is important in assuring the model validity. The HTA researcher could further improve the model and its parameters through consulting other stakeholders including policy makers, professional groups, and relevant patients. However, there are a few concerns regarding stakeholder involvement in an early HTA. Confidential information disclosure and conflict of interest were among major issues as the innovator may not be willing to share information to other stakeholders or require certain administrative processes, for example, signing a nondisclosure agreement before the stakeholder consultation can take place. In our setting, this kind of procedure has not been well-established and the wider group of stakeholders is not familiar. The government, innovators and HTA researchers should work together to standardize, ideally simplify, these protocols to overcome the aforementioned challenges.

The limitations faced during the study show the importance of long-term collaboration between HTA researchers and innovators. Models are not static and can be improved, and more accurate data can be located and used. A long-term collaboration also allows the involvement of external panels and acquiring the most suitable data source. The current model and analysis can serve as a starting point for the innovator to plan their next steps and facilitate the discussion with relevant stakeholders. By continuously updating the model with new information that arises, the model at the early stage can serve as an intermediate model for the final model in the traditional HTA analysis. As such, for this study, the innovator and the HTA researcher agreed to meet again after the first clinical trial to update the results. The innovator was also recommended to consider horizon scanning systems to identify potential competitors and to examine patient preference in the clinical trial, for example, conjoint analysis.

## Conclusions

Early HTA was conducted to examine a soft robotic sock in poststroke patients. Our results show the new technology is expected to be cost-saving, and suggest the directions to improve the technology and the data to be collected in the future clinical trial. Practical steps were provided for the researchers and innovators to conduct similar works. Challenges of the collaboration were highlighted. Early HTA is helpful in guiding medical innovation development, but its application among R&D communities is low. Real-world collaboration can raise the awareness of early HTA among R&D communities and promote the application of early HTA.

## Supplementary material

For supplementary material accompanying this paper visit https://doi.org/10.1017/S026646232200335X. click here to view supplementary material
