# Supplementary material for: Using early health economic modeling to inform medical innovation development: a soft robotic sock in poststroke patients in Singapore
Source: Int J Technol Assess Health Care. 2023 Jan 11;39(1):e4. doi: 10.1017/S026646232200335X (PMC11574750; doi:10.1017/S026646232200335X)
Supplement: Supplementary file 1 [file S026646232200335Xsup001.docx]

USING EARLY HEALTH ECONOMIC MODELLING TO INFORM MEDICAL INNOVATION DEVELOPMENT: A SOFT ROBOTIC SOCK IN POST-STROKE PATIENTS IN SINGAPORE

**Appendix**

Table S1: Parameter Values

| **Parameter names** | **Explanation** | **Mean** | **Distribution** | **Range in DSA** | **Source** |
| --- | --- | --- | --- | --- | --- |
| prob_compliance_ipc | Compliance rate of IPC | 0.74 | Fixed | NA | Clinical team |
| prob_compliance_vac | Compliance rate of VACOM | 0.8 | Beta | 0.67-0.90 | Clinical team |
| prob_dvt | Incidence of DVT with no IPC nor VACOM | 0.121 | Beta | 0.077-0.173 | [1] |
| rr_dvt_ipc | Relative risk (RR) of DVT: IPC vs no IPC | 0.702 | Log normal | 0.47-1.01 | [1] |
| rr_dvt_vac_ipc | RR of DVT: VACOM vs IPC | 0.9 | Log normal | 0.84-0.96 | Innovator |
| prob_pe_nodvt | Incidence of PE for patients with no DVT | 0.0051 | Beta | 0.0033-0.0073 | [2] |
| rr_pe_withdvt | RR of PE: DVT vs no DVT | 52.49 | Log normal | 34.95-76.13 | [2] |
| prob_death_dis | Death rate at discharge for patients with no DVT nor PE: IS (HA) | 0.0395 (0.2317) | Beta | 0.0252-0.0563  (0.1474-0.3282) | [3] |
| rr_death_dis_dvt | RR of death at discharge: DVT vs no DVT nor PE | 1 | Fixed | NA | Clinical team |
| rr_death_dis_pe | RR of death at discharge: PE vs no DVT nor PE | 1.896 | Log normal | 1.265-2.751 | [2] |
| prob_death_postdis | Death rate post discharge for patients with no DVT nor PE: IS (HA) | 0.0198 (0.0209) | Beta | 0.0127-0.0284  (0.0134-0.0297) | [3] |
| rr_death_postdis_dvt | RR of death post discharge: DVT vs no DVT nor PE | 1 | Fixed | NA | Clinical team |
| rr_death_postdis_pe | RR of death post discharge: PE vs no DVT nor PE | 2.264 | Log normal | 1.507-3.292 | [2] |
| prob_recdvt | Incidence of recurrent DVT for patients with no PE | 0.0293 | Beta | 0.0188-0.0418 | [4] |
| rr_recdvt_pe | RR of recurrent DVT: PE vs no PE | 1.6 | Log normal | 1.059-2.318 | [4] |
| prob_recpe | Incidence of PE post discharge for patients with DVT | 0.0088 | Beta | 0.0057-0.0126 | [4] |
| rr_recpe_pe | RR of PE post discharge: PE vs DVT | 4 | Log normal | 2.66-5.79 | [4] |
| prob_recstr | Incidence of recurrent stroke | 0.031 | Beta | 0.020-0.044 | [5] |
| prob_ankcon | Incidence of ankle contracture | 0.25 | Beta | 0.156-0.354 | [6] |
| rr_ankcon_vac | RR of ankle contracture: VACOM vs IPC + manual ankle movement | Optimistic: 0.3  Conservative: 0.9 | Log normal | Optimistic: 0.072-0.84 Conservative: 0.84-0.96 | Clinical team |
| cost_vac | Additional cost per patients: VACOM vs IPC + manual ankle movement | 150 | Fixed | NA | Innovator |
| cost_screen_dvt | Screening cost DVT | 209 | Fixed | NA | Clinical team |
| cost_screen_pe | Screening cost PE | 321 | Fixed | NA | Clinical team |
| cost_treat_str | Cost of inpatient treatment: stroke: IS (HA) | 8631.07 (18664.67) | Log normal | 344.87-44473.94  (782.75-96831.01) | [7] |
| cost_treat_dvt | Cost of inpatient treatment due to DVT | 4450.72 | Log normal | 2946.76-6390.35 | [1] |
| cost_treat_pe | Cost of inpatient treatment due to PE | 20113.58 | Log normal | 13337.34-29377.95 | [1] |
| cost_treat_dvtpe | Cost of inpatient treatment due to PE + DVT | 20113.58 | Log normal | 13337.34-29377.95 | [1] |
| cost_treat_str_fol | Cost of follow-up treatment stroke: IS (HA) | 867.94 (804.95) | Log normal | 355.39-1755.74  (568.13-1107.86) | [7] |
| cost_treat_str_rec | Cost of recurrent stroke treatment | 2007.39 | Log normal | 20.00-12395.51 | [7] |
| cost_treat_dvt_rec | Cost of recurrent DVT treatment | 4450.72 | Log normal | 2948.51-6460.73 | [1] |
| cost_treat_pe_rec | Cost of recurrent PE treatment | 20113.58 | Log normal | 13405.70-29152.42 | [1] |
| cost_postdis_death | Cost of death post discharge: IS (HA) | 433.97 (402.48) | Log normal | 179.58-895.53  (283.19-557.02) | [7] |
| cost_treat_ankcon | Cost of ankle contracture treatment | 4843.22 | Log normal | 3231.42-7015.64 | Clinical team |
| qaly_ndnp_1m_death | QALY: patient without DVT without PE die at discharge | 0.0258 | Normal | 0.0245-0.0271 | [8] |
| qaly_ndwp_1m_death | QALY: patient without DVT with PE die at discharge | 0.0196 | Normal | 0.0186-0.0206 | [8, 9] |
| qaly_wdnp_1m_death | QALY: patient with DVT without PE die at discharge | 0.0227 | Normal | 0.0216-0.0238 | [8, 10] |
| qaly_wdwp_1m_death | QALY: patient with DVT with PE die at discharge | 0.0196 | Normal | 0.0186-0.0206 | [8–10] |
| qaly_ndnp_1_12m_well | QALY: without DVT without PE during inpatient period, no complication after discharge | 0.68 | Normal | 0.6462-0.7130 | [8] |
| qaly_ndnp_1_12m_rstr | QALY: without DVT without PE during inpatient period, with recurrent stroke after discharge | 0.6333 | Normal | 0.6024-0.6644 | [8] |
| qaly_ndnp_1_12m_ankcon | QALY: without DVT without PE during inpatient period, with ankle contracture after discharge | 0.6182 | Normal | 0.5875-0.6484 | [8, 11] |
| qaly_ndnp_1_12m_death | QALY: without DVT without PE during inpatient period, die after discharge | 0.3167 | Normal | 0.3016-0.3323 | [8] |
| qaly_ndwp_1_12m_well | QALY: without DVT with PE during inpatient period, no complication after discharge | 0.5527 | Normal | 0.5259-0.5794 | [8, 9] |
| qaly_ndwp_1_12m_rstr | QALY: without DVT with PE during inpatient period, with recurrent stroke after discharge | 0.5142 | Normal | 0.4892-0.5398 | [8, 9] |
| qaly_ndwp_1_12m_rpe | QALY: without DVT with PE during inpatient period, with recurrent PE after discharge | 0.5418 | Normal | 0.5152-0.5682 | [8, 9] |
| qaly_ndwp_1_12m_ankcon | QALY: without DVT with PE during inpatient period, with ankle contracture after discharge | 0.5527 | Normal | 0.5259-0.5794 | [8, 9] |
| qaly_ndwp_1_12m_death | QALY: without DVT with PE during inpatient period, die after discharge | 0.2526 | Normal | 0.2403-0.2646 | [8, 9] |
| qaly_wdnp_1_12m_well | QALY: with DVT without PE during inpatient period, no complication after discharge | 0.5969 | Normal | 0.5674-0.6256 | [8, 10] |
| qaly_wdnp_1_12m_rstr | QALY: with DVT without PE during inpatient period, with recurrent stroke after discharge | 0.5559 | Normal | 0.5284-0.5831 | [8, 10] |
| qaly_wdnp_1_12m_rdvt | QALY: with DVT without PE during inpatient period, with recurrent DVT after discharge | 0.5969 | Normal | 0.5674-0.6256 | [8, 10] |
| qaly_wdnp_1_12m_rpe | QALY: with DVT without PE during inpatient period, with recurrent PE after discharge | 0.5672 | Normal | 0.5393-0.5947 | [8, 10] |
| qaly_wdnp_1_12m_ankcon | QALY: with DVT without PE during inpatient period, with ankle contracture after discharge | 0.5795 | Normal | 0.5510-0.6076 | [8, 10, 11] |
| qaly_wdnp_1_12m_death | QALY: with DVT without PE during inpatient period, die after discharge | 0.2780 | Normal | 0.2642-0.2916 | [8, 10] |
| qaly_wdwp_1_12m_well | QALY: with DVT with PE during inpatient period, no complication after discharge | 0.5527 | Normal | 0.5250-0.5804 | [8–10] |
| qaly_wdwp_1_12m_rstr | QALY: with DVT with PE during inpatient period, with recurrent stroke after discharge | 0.5142 | Normal | 0.4893-0.5392 | [8–10] |
| qaly_wdwp_1_12m_rdvt | QALY: with DVT with PE during inpatient period, with recurrent DVT after discharge | 0.5527 | Normal | 0.5259-0.5799 | [8–10] |
| qaly_wdwp_1_12m_rpe | QALY: with DVT with PE during inpatient period, with recurrent PE after discharge | 0.5418 | Normal | 0.5149-0.5684 | [8–10] |
| qaly_wdwp_1_12m_ankcon | QALY: with DVT with PE during inpatient period, with ankle contracture after discharge | 0.5527 | Normal | 0.5250-0.5799 | [8–10] |
| qaly_wdwp_1_12m_death | QALY: with DVT with PE during inpatient period, die after discharge | 0.2526 | Normal | 0.2403-0.2650 | [8–10] |

Note: DSA: deterministic sensitivity analysis; IPC, intermittent pneumatic compression; VACOM, Venous Assistance and Contracture Management; ROM, range of motion; DVT, deep vein thrombosis; RR, relative risk; PE, pulmonary embolism; IS, ischemic stroke; HA, haemorrhagic stroke; QALY, quality-adjusted life year.

Commonly used distributions were selected for the parameters. The ranges for DSA were the 95% confidence interval generated using simulation with 10000 times.

Table S2: Deterministic Cost-Utility Analysis

|  | Incremental cost (S$) | Incremental QALY | ICER |
| --- | --- | --- | --- |
| Optimistic scenario: relative risk of ankle contracture 0.3 | | | |
| IS patients | -581.62 | 0.0084 | -68,713.60 |
| AH patients | -446.09 | 0.0070 | -63,760.38 |
| Conservative scenario: relative risk of ankle contracture 0.9 | | | |
| IS patients | -23.96 | 0.0018 | -12,873.43 |
| AH patients | -2.88 | 0.0017 | -1680.06 |

Note: IS, ischemic stroke; HA, haemorrhagic stroke; QALY, quality-adjusted life year; ICER, incremental cost-effectiveness ratio.

Table S3: List of Parameters that Having High Impact on the Results

| Parameter | Optimistic scenario | | | | Conservative scenario | | | |
| --- | --- | --- | --- | --- | --- | --- | --- | --- |
|  | IS patient | | HA patient | | IS patient | | HA patient | |
|  | DSA | EVPPI | DSA | EVPPI | DSA | EVPPI | DSA | EVPPI |
| RR of ankle contracture: VACOM vs IPC + manual movement | Y | Y | Y | Y | Y | Y | Y | Y |
| RR of DVT: VACOM vs IPC | Y |  | Y | Y | Y | Y | Y | Y |
| Compliance rate VACOM | Y | Y | Y |  | Y | Y | Y | Y |
| Probability of ankle contracture in the control group | Y | Y | Y | Y | Y | Y | Y | Y |
| Probability of death at discharge for patients with no DVT nor PE | Y |  | Y | Y |  |  |  |  |
| RR of death at discharge: PE vs no DVT nor PE |  |  | Y |  |  |  |  |  |
| RR of PE: DVT vs no DVT |  |  |  |  | Y | Y | Y | Y |
| RR of death post discharge: PE vs no DVT nor PE |  |  |  |  |  |  | Y |  |
| Probability of DVT with no IPC nor VACOM | Y |  | Y |  | Y | Y | Y | Y |
| Probability of PE for patients with no DVT |  |  |  |  |  | Y | Y | Y |
| Cost of treatment: ankle contracture |  |  |  | Y |  | Y |  | Y |
| QALY: without DVT without PE during inpatient period, no complication after discharge | Y | Y | Y | Y | Y | Y | Y | Y |
| QALY: without DVT without PE during inpatient period, with ankle contracture after discharge | Y | Y | Y | Y | Y | Y |  | Y |
| QALY: with DVT without PE during inpatient period, with ankle contracture after discharge | Y |  | Y |  | Y |  | Y |  |
| QALY: with DVT without PE during inpatient period, no complication after discharge | Y |  |  |  | Y |  |  |  |

Notes: “Y” indicates the parameter is among the top 10 parameters in terms of impact.

IS, ischemic stroke; HA, haemorrhagic stroke; EVPPI, expected value of partial perfect information; RR, relative risk; VACOM, Venous Assistance and Contracture Management; IPC, intermittent pneumatic compression; DVT, deep vein thrombosis; PE, pulmonary embolism; QALY, quality-adjusted life year.

Figure S1: Relationship between Price Premium and ICER


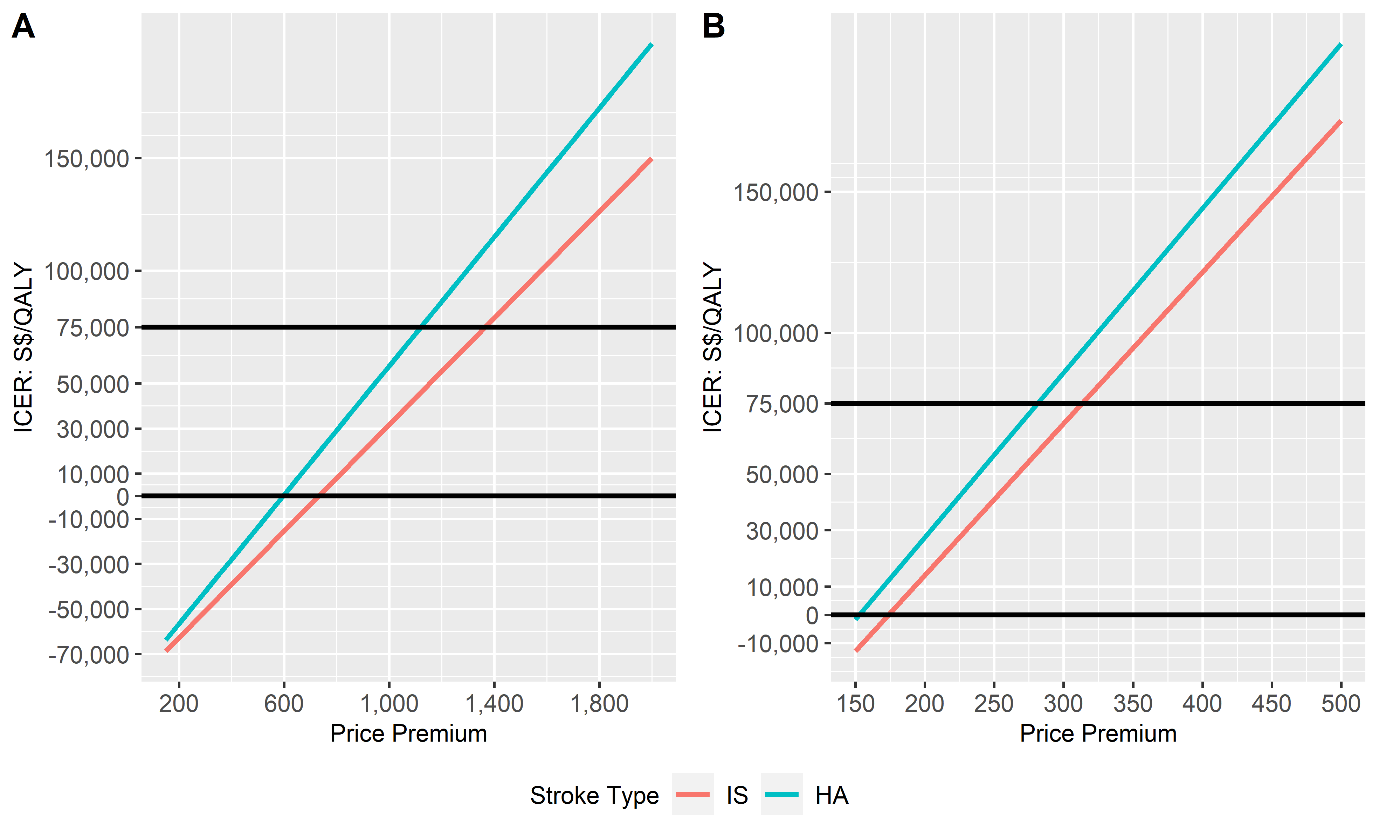


Notes: Panel A: optimistic scenario; Panel B: conservative scenario.

IS, ischemic stroke; HA, haemorrhagic stroke; QALY, quality-adjusted life year; ICER, incremental cost-effectiveness ratio.

Figure S2: Cost-effectiveness Acceptability Curve


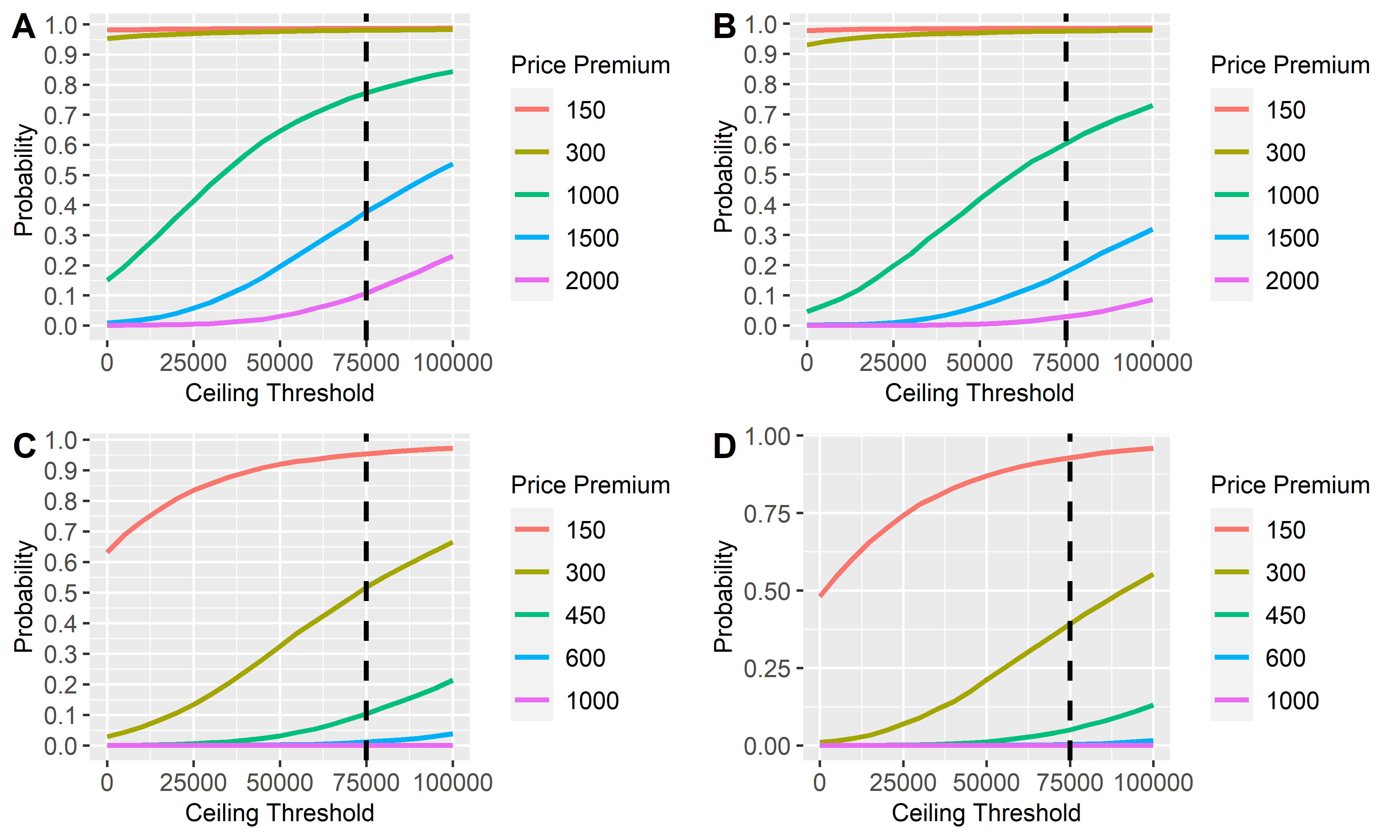


Notes: Panel A: Ischemic stroke patients under the optimistic scenario; Panel B: Haemorrhagic stroke patients under the optimistic scenario; Panel C: Ischemic stroke patients under the conservative scenario; Panel D: Haemorrhagic stroke patients under the conservative scenario.

Figure S3: Threshold Analysis – Price Premium vs RR of Ankle Contracture


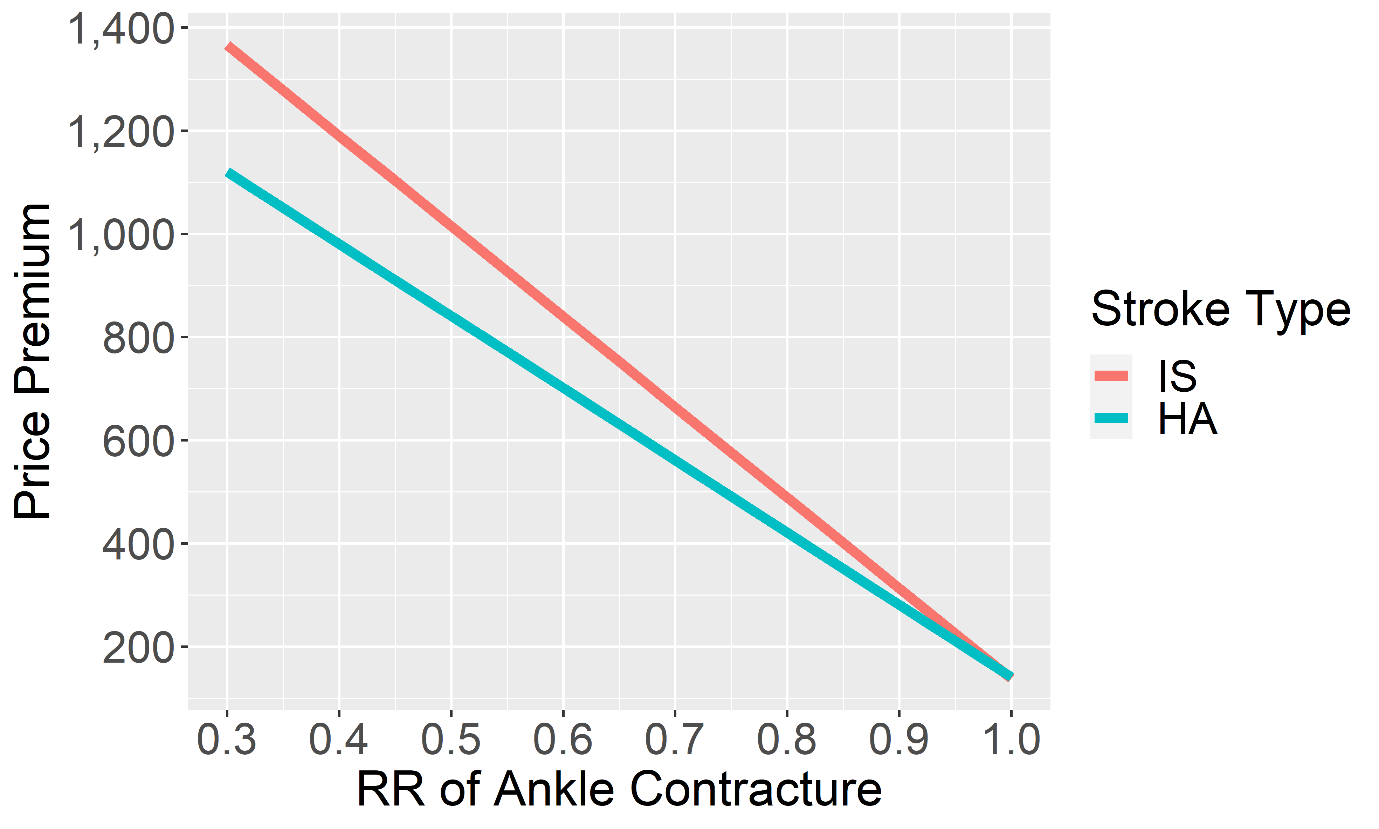


Notes: IS, ischemic stroke; HA, haemorrhagic stroke; RR, relative risk.

Figure S4: Threshold Analysis – Price Premium vs Compliance Rate of VACOM


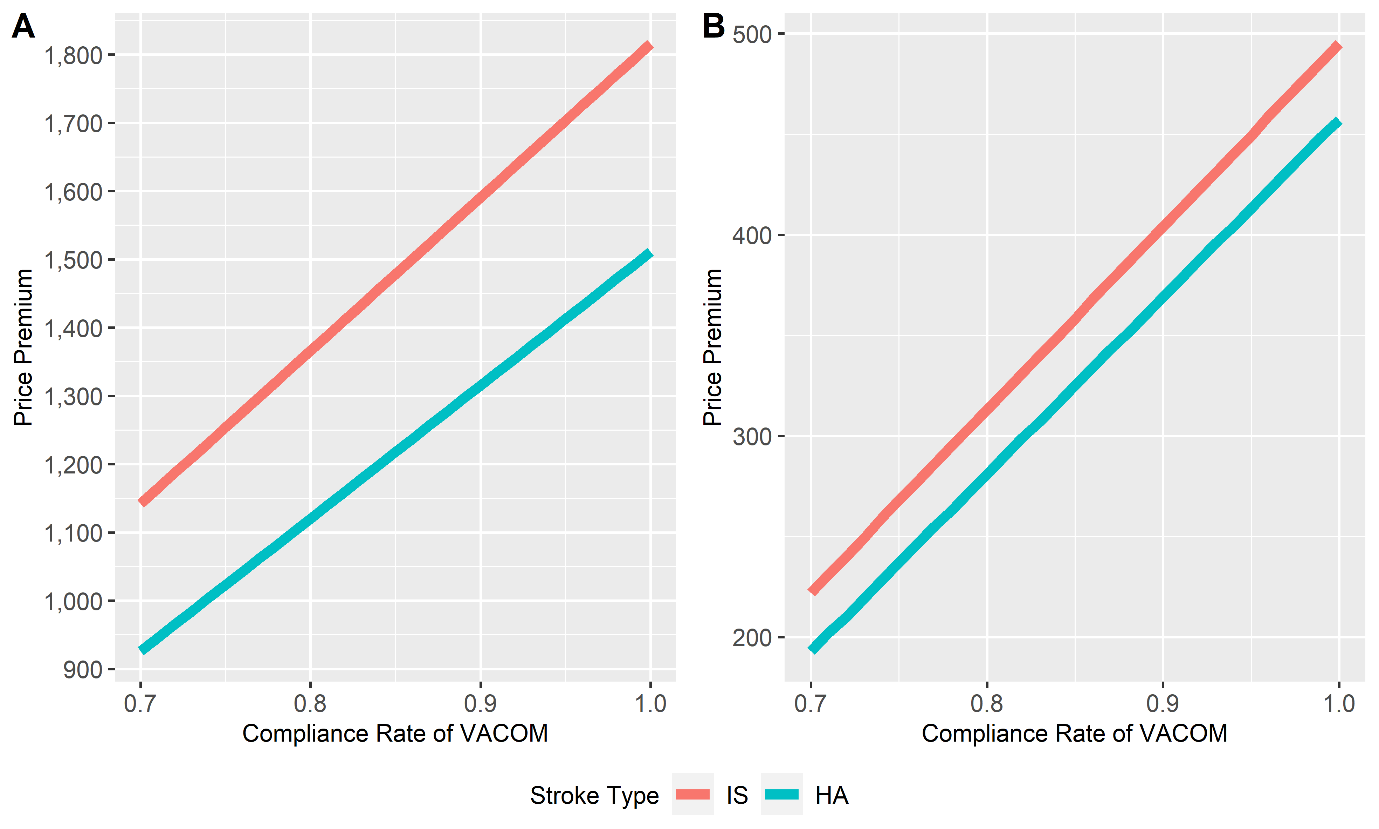


Notes: Panel A: optimistic scenario; Panel B: conservative scenario.

IS, ischemic stroke; HA, haemorrhagic stroke; VACOM, Venous Assistance and Contracture Management

Figure S5: Threshold Analysis – Price Premium vs Incidence Rate of Ankle Contracture


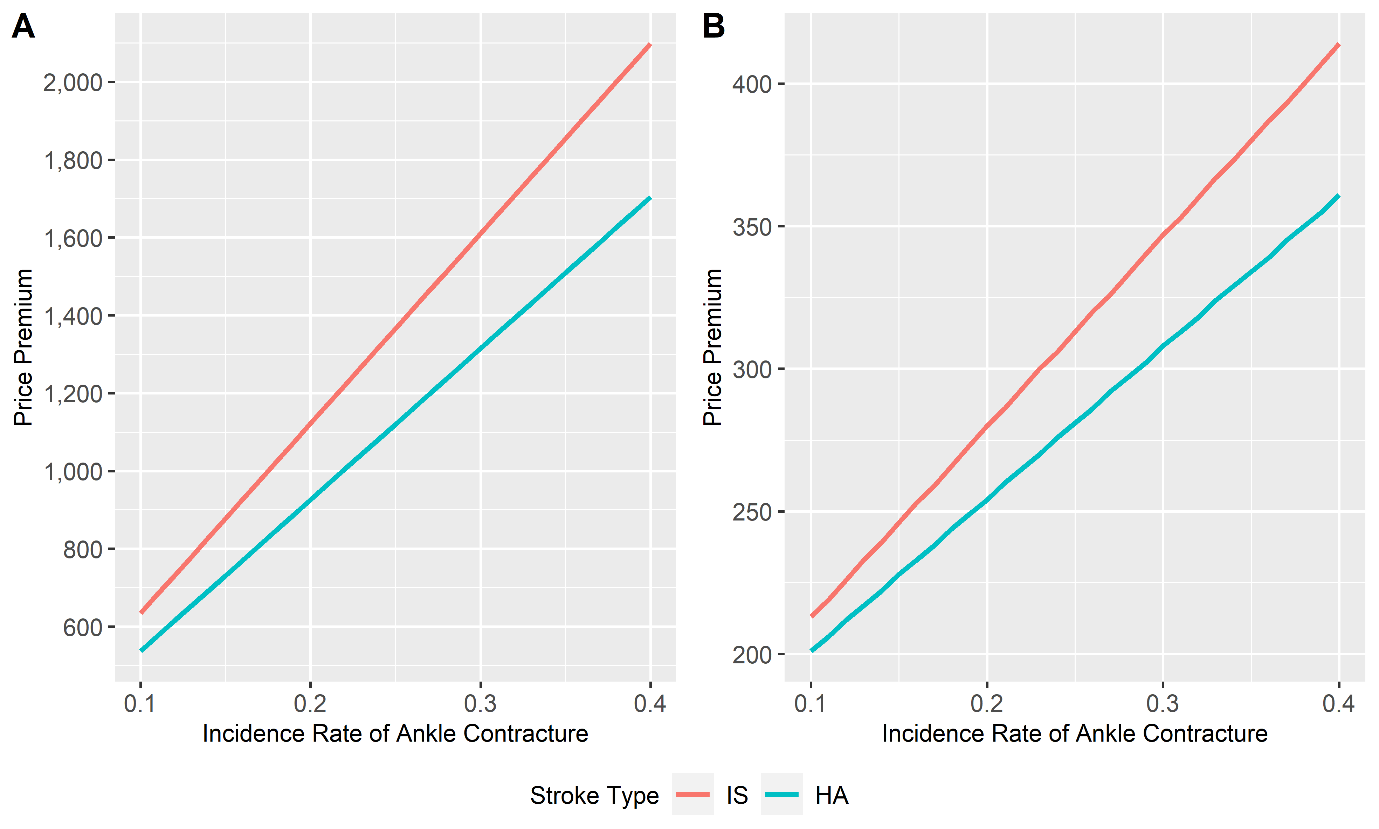


Notes: Panel A: optimistic scenario; Panel B: conservative scenario.

IS, ischemic stroke; HA, haemorrhagic stroke.

Figure S6: EVPI Curves


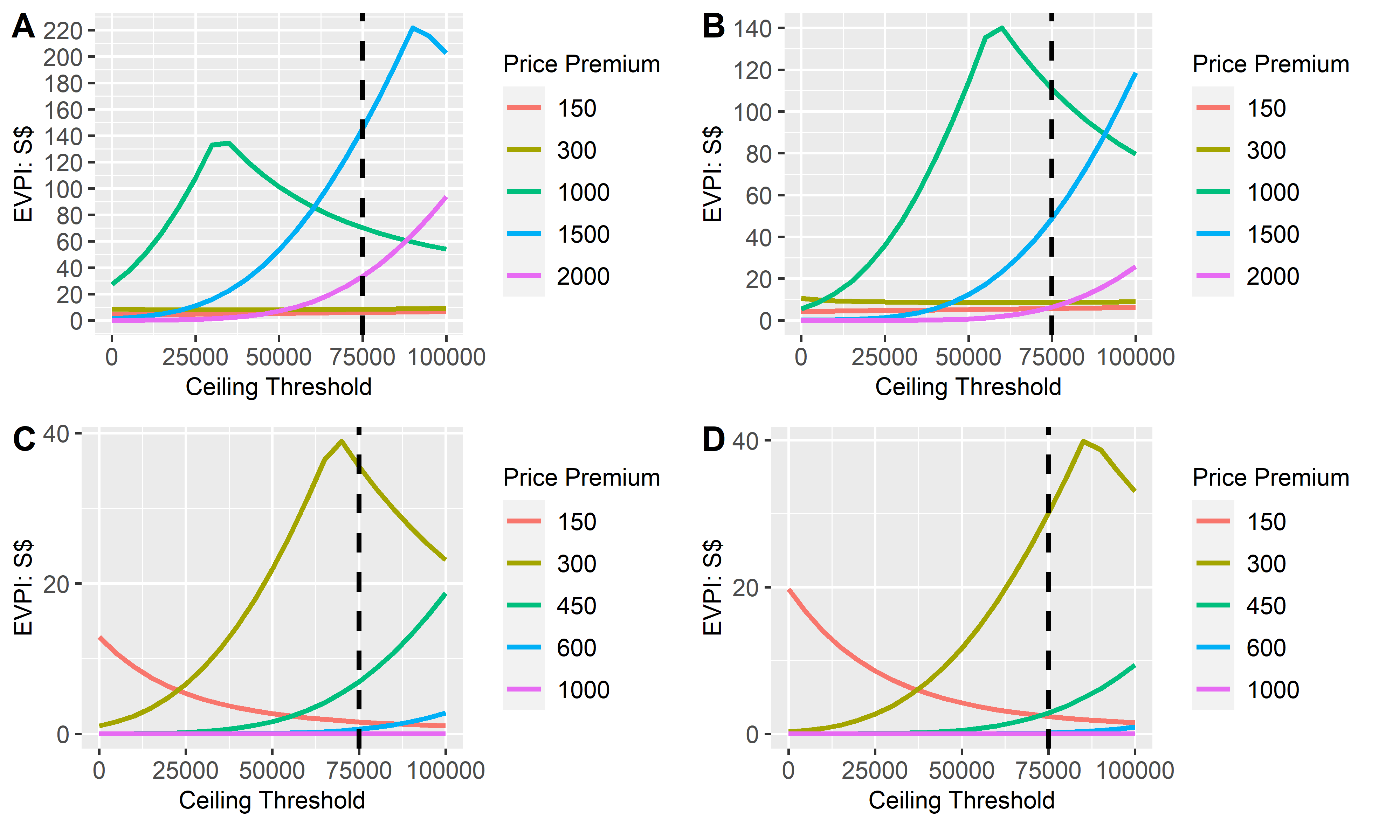


Notes: Panel A: Ischemic stroke patients under the optimistic scenario; Panel B: Haemorrhagic stroke patients under the optimistic scenario; Panel C: Ischemic stroke patients under the conservative scenario; Panel D: Haemorrhagic stroke patients under the conservative scenario.

EVPI, expected value of perfect information.

Figure S7: EVPPI


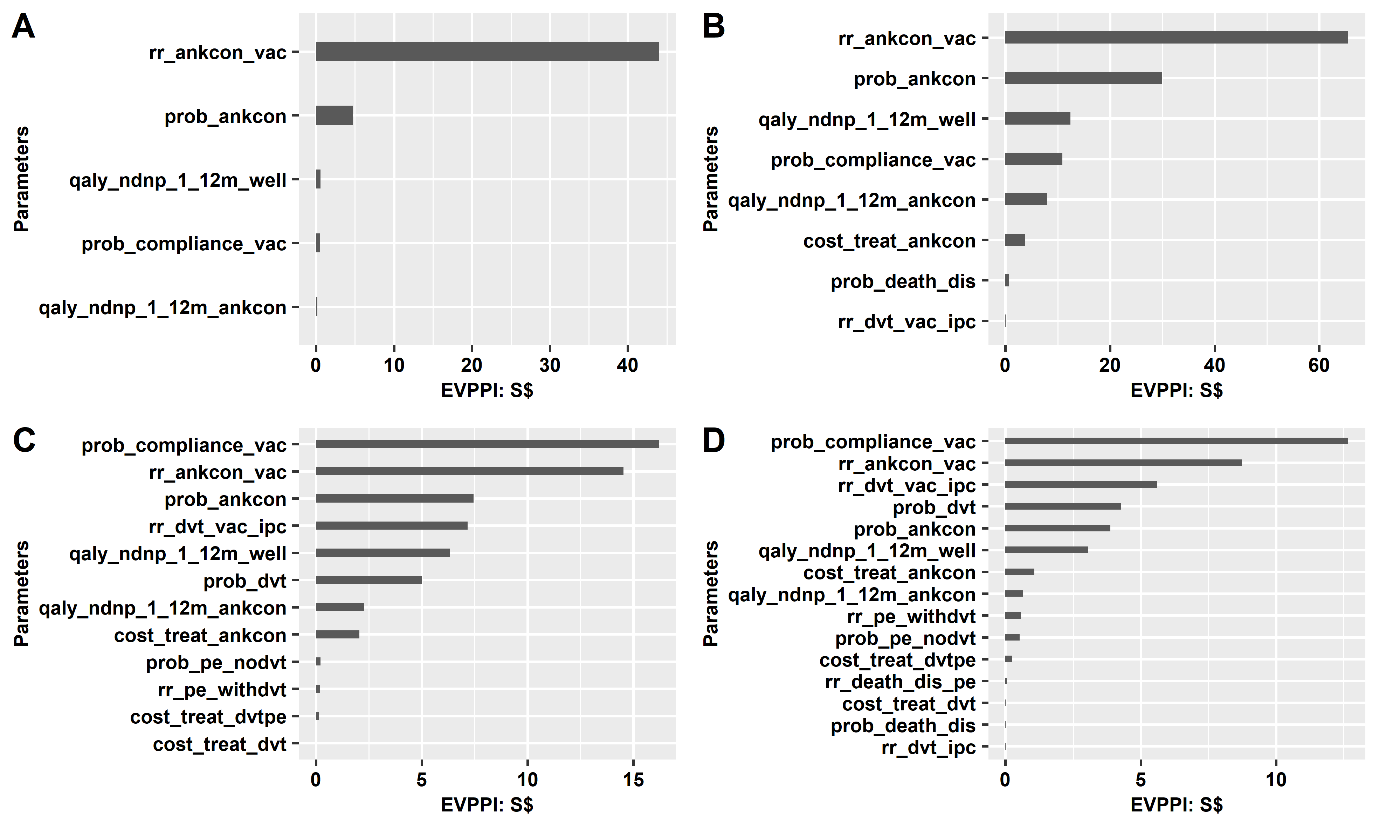


Notes: Panel A: Ischemic stroke patients under the optimistic scenario; Panel B: Haemorrhagic stroke patients under the optimistic scenario; Panel C: Ischemic stroke patients under the conservative scenario; Panel D: Haemorrhagic stroke patients under the conservative scenario.

EVPPI, expected value of partial perfect information.

Readers can refer to Table 1 for the full details of each parameter.

**Model Assumptions**

VACOM:

- VACOM is expected to affect the incidence of DVT, but not to affect the incidence of PE directly. It can affect the incidence of PE through the effect on the incidence of DVT.
- Conditioning on the impact on the incidence of DVT, VACOM has no direct effect on the mortality, recurring stroke, recurring DVT, and recurring PE.
- VACOM is expected to improve ankle ROM and decrease the incidence of ankle contracture.

IPC:

- IPC is expected to affect the incidence of DVT, but not to affect the incidence of PE directly. It can affect the incidence PE through the effect on the incidence of DVT.
- Conditioning on the impact on the incidence of DVT, IPC has no direct effect on the mortality, recurring stroke, recurring DVT, and recurring PE.
- IPC has no impact on ROM nor ankle contracture.

Stroke

- Mortality at discharge, mortality post-discharge, and direct medical cost are different between ischemic stroke and haemorrhagic stroke. The remaining factors, e.g., impact of VACOM, are the same between ischemic stroke and haemorrhagic stroke.

DVT and PE

- DVT affects the incidence of PE. Conditioning on the effect on PE, DVT has no direct effect on mortality.
- DVT affects incidence of recurring DVT and recurring PE.
- PE affects the mortality at discharge and after discharge.
- PE affects the incidence of recurring DVT and recurring PE.
- Neither DVT nor PE has any effect on the development of recurring stroke nor ankle contracture.
- For the patients with PE+DVT, we use the same epidemiology parameters and cost parameters as patients with only PE.

Mortality

- Mortality of stroke patients within 30 days are used when calculating the mortality at discharge, and mortality of stroke patients from 30 days to 1 year are used when calculating the mortality after discharge.
- Death at discharge is assumed to happen at 15^th^ day on average. Death after discharge is assumed to happen at the start of the 7^th^ month on average.

Recurring stroke, recurring DVT, recurring PE, and ankle contracture

- We assume all these will happen at the start of 7^th^ month on average.
- For each patient, we assume at most one of the recurring conditions will happen.
- For recurring condition, we do not consider further comorbidities and mortality, e.g.: for recurring stroke, we no longer consider the subsequent DVT.

Cost: Stroke

- Stroke cost are 1 year cost post-stroke from local studies, capturing inpatients and outpatients after discharge.

Cost: DVT and PE

- For the additional cost due to DVT+PE, we assume it is the same as the additional cost due to PE.
- The cost of recurring DVT is the same as the cost of DVT during the inpatient period.
- The cost of recurring PE is the same as the cost of PE during the inpatient period.

Utility:

- We used the multiplication formula for comorbidities: i.e. utility(stroke+DVT) = utility(stroke)*utility(DVT).
- For patients with more than 1 comorbidities, we consider the comorbidity that leading to the lowest QALY. For example, for patients with DVT and PE, we considered the utility loss from PE: utility(stroke+DVT+PE) = utility(stroke)*utility(PE).
- For patients with recurring stroke and recurring PE, we re-adjust the utility at 7^th^ month to 1^st^ month as the utility scores are available at multiple time points after discharge.
- Improved ankle ROM does not affect utility directly. It can affect utility indirectly through the reduced risk of ankle contracture.

Conventional distributions were assumed for the corresponding parameters. The standard deviation were obtained from the literature if available. Otherwise, the standard deviation were assumed as the follows:

- prob_compliance_vac (0.8), rr_dvt_vac_ipc (0.9), rr_ankcon_vac (0.9)
  - SE= (1-mean)*0.3
- QALY parameters: The differences in QALYs are very small across different health states. Smaller standard deviations for QALYs were assumed to avoid the scenarios that sick patients having higher utility than healthy patients.
  - SE = mean*0.025
- Remaining parameters
  - SE = mean*0.2

Note: IPC, intermittent pneumatic compression; VACOM, Venous Assistance and Contracture Management; ROM, range of motion; DVT, deep vein thrombosis; RR, relative risk; PE, pulmonary embolism; IS, ischemic stroke; HA, haemorrhagic stroke; QALY, quality-adjusted life year; SE, standard error.

Cost and Benefit of Conducting Additional Research

The expected cost of a clinical trial, aiming for 100 participants and lasting for 2 years, is round S$1.44 million.

The manpower cost is around S$0.7 million, including researchers, and project and business development managers. Variable cost that depends on the sample size of the trial is round S$0.2 million, including ultrasound, physiotherapist and allowance for all the patients. The next major cost component is the outsourced manufacturing of clinical-grade soft robotic sock units, which is expected to be S$0.12 million. The remaining cost include utilities, space, and other miscellaneous expenses.

There were 8326 stroke patients in Singapore in year 2018 based on Singapore Stroke Registry Annual Report 2018.[12] However, the exact number of patients that satisfy the inclusion criteria of our study is unknown. We assumed that 40 percent (3330) of the stroke patients, who are with moderate to severe stroke, can benefit from the innovation. [13] We consider a 10-years time horizon and a discount rate of 3 percent.[14] A minimum EVPI of S$49.3 is required to make the population-EVPI equal or bigger than the research cost of S$1.44 million. Hence, the research can be justified if the innovator wants to set the price premium between S$1000 and S$1500 under the optimistic scenario at the ceiling threshold of S$75,000. The research cannot be justified under the conservative scenarios, when the clinical effectiveness of the innovation is low.

Additional opportunity cost from the research include: 1) the 100 participants may not benefit from the best treatment; 2) the best treatment will be delayed by 2 years. Considering these, a minimum EVPI of S$52.4 is required. However, the number of stroke patients is expected to increase in the future. Without the clinical trial, the decision-making process could take longer which will delay the optimal treatment as well. EVPI gives the theoretical upper bound of the benefit per patient from research. EVSI can be further considered to optimize the clinical trial in practice. However, one of the major aims of the coming clinical trial is to demonstrate the clinical efficacy of the innovation. Hence, the innovator was recommended to consider the sample size focusing on the statistical power. More weight should be given to the results from VOI when designing subsequent clinical trials.

References:

[1] Dennis M, Sandercock P, Graham C, et al. The Clots in Legs or sTockings after Stroke (CLOTS) 3 trial: A randomised controlled trial to determine whether or not intermittent pneumatic compression reduces the risk of post-stroke deep vein thrombosis and to estimate its cost-effectiveness. *Health Technol Assess (Rockv)*; 19. Epub ahead of print 1 September 2015. DOI: 10.3310/hta19760.

[2] Pongmoragot J, Rabinstein AA, Nilanont Y, et al. Pulmonary embolism in ischemic stroke: clinical presentation, risk factors, and outcome. *J Am Heart Assoc*; 2. Epub ahead of print 2013. DOI: 10.1161/JAHA.113.000372.

[3] National Registry of Diseases Office. Singapore Stroke Registry Annual Report 2017. 2019; 2: 1–66.

[4] Eichinger S, Weltermann A, Minar E, et al. Symptomatic Pulmonary Embolism and the Risk of Recurrent Venous Thromboembolism. *Arch Intern Med* 2004; 164: 92–96.

[5] Sun Y, Lee SH, Heng BH, et al. 5-year survival and rehospitalization due to stroke recurrence among patients with hemorrhagic or ischemic strokes in Singapore. *BMC Neurol*; 13. Epub ahead of print 3 October 2013. DOI: 10.1186/1471-2377-13-133.

[6] Kwah LK, Harvey LA, Diong JHL, et al. Half of the adults who present to hospital with stroke develop at least one contracture within six months: An observational study. *J Physiother* 2012; 58: 41–47.

[7] Ng CS, Toh MPHS, Ng J, et al. Direct medical cost of stroke in Singapore. *Int J Stroke* 2015; 10: 75–82.

[8] Yeoh YS, Koh GCH, Tan CS, et al. Health-related quality of life loss associated with first-time stroke. *PLoS One*; 14. Epub ahead of print 1 January 2019. DOI: 10.1371/journal.pone.0211493.

[9] Chuang LH, Gumbs P, van Hout B, et al. Health-related quality of life and mortality in patients with pulmonary embolism: a prospective cohort study in seven European countries. *Qual Life Res* 2019; 28: 2111–2124.

[10] Utne KK, Tavoly M, Wik HS, et al. Health-related quality of life after deep vein thrombosis. *Springerplus*; 5. Epub ahead of print 1 December 2016. DOI: 10.1186/s40064-016-2949-z.

[11] Gillard PJ, Sucharew H, Kleindorfer D, et al. The negative impact of spasticity on the health-related quality of life of stroke survivors: A longitudinal cohort study. *Health Qual Life Outcomes*; 13. Epub ahead of print 29 September 2015. DOI: 10.1186/s12955-015-0340-3.

[12] National Registry of Diseases Office. Singapore Stroke Registry Annual Report 2018. *Singapore Stroke Regist Natl Regist Dis Off* 2020; 2: 66.

[13] Tyagi S, Koh GCH, Nan L, et al. Healthcare utilization and cost trajectories post-stroke: role of caregiver and stroke factors. *BMC Health Serv Res* 2018; 18: 881.

[14] Agency for Care Effectiveness Singapore. Drug evaluation methods and process guide, http://www.ace-hta.gov.sg/public-data/our-process-and-methods/ACE methods and process guide for drug evaluation (5 Feb 2018).pdf (2018).
